# The dependency on central government funding of decentralised health systems: experiences of the challenges and coping strategies in the Kongwa District, Tanzania

**DOI:** 10.1186/1472-6963-14-39

**Published:** 2014-01-25

**Authors:** Gasto Frumence, Tumaini Nyamhanga, Mughwira Mwangu, Anna-Karin Hurtig

**Affiliations:** 1 Department of Development Studies, School of Public Health and Social Sciences, Muhimbili University of Health and Allied Sciences, box 65454, Dar es Salaam, Tanzania; 2 Epidemiology and Global Health, Department of Public Health and Clinical Medicine, Umeå University, SE-901 85, Umeå, Sweden

**Keywords:** Decentralisation, Health systems, Late disbursement, Central government, Local authorities, Tanzania

## Abstract

**Background:**

Decentralised health systems in Tanzania depend largely on funding from the central government to run health services. Experience has shown that central funding in a decentralised system is not an appropriate approach to ensure the effective and efficient performance of local authorities due to several limitations. One of the limitations is that funds from the central government are not disbursed on a timely basis, which in turn, leads to the serious problem of shortage of financial resources for Council Health Management Teams (CHMT). This paper examines how dependency on central government funding in Tanzania affects health activities in Kongwa district council and the strategies used by the CHMT cope with the situation.

**Methods:**

The study adopted a qualitative approach and data were collected using semi-structured interviews and focus group discussions. One district in the central region of Tanzania was strategically selected. Ten key informants involved in the management of health service delivery at the district level were interviewed and one focus group discussion was held, which consisted of members of the council health management team. The data generated were analysed for themes and patterns.

**Results:**

The results showed that late disbursement of funds interrupts the implementation of health activities in the district health system. This situation delays the implementation of some activities, while a few activities may not be implemented at all. However, based on their prior knowledge of the anticipated delays in financial disbursements, the council health management team has adopted three main strategies to cope with this situation. These include obtaining supplies and other services on credit, borrowing money from other projects in the council, and using money generated from cost sharing.

**Conclusion:**

Local government authorities (LGAs) face delays in the disbursement of funds from the central government. This has necessitated introduction of informal coping strategies to deal with the situation. National-level policy and decision makers should minimise the bureaucracy involved in allocating funds to the district health systems to reduce delays.

## Background

The health system in Tanzania is organised into three components: the district component (the district hospital, health centres, dispensaries, and community health services), secondary and tertiary hospitals and other tertiary-level institutions (teaching institutions), and the central level, which provides support services such as policy-making, the development of guidelines and standards, donor coordination, and monitoring and evaluation [1]. Like many other developing countries, the district (council) health system in Tanzania is the key structure for the delivery of basic health services to the majority of the population. However, the district health system is suffering from various constraints in its delivery of health services to the people. Among others, these constraints include the lack of adequate resources (particularly funds), the shortage of qualified staff, which leads to poor planning and poor management of the available resources, and corruption [2].

Some developing countries, including Tanzania, have put into place various reforms aimed at reorganising both the central and the local government structures in order to enhance the efficiency and effectiveness of the delivery of social services to communities. Health sector reforms (HSR) were introduced in Tanzania in the early 1990s and the district health system was identified as the focal point for the operationalisation of these reforms. The district health system was selected mainly because it is the structure that is the closest to communities and it is where most of the essential health services are provided [1].

The government of Tanzania introduced the decentralisation policy, which was the key component of both health sector and local government reforms. In the health sector, the decentralisation policy was aimed at devolving the provision of health services to Local Government Authorities (LGAs). Furthermore, the decentralisation by devolution (D by D) policy was purposively introduced in order to increase the mandate of LGAs in making decisions regarding how health care services are delivered at this level, and to encourage the active participation of the users of the services [3]. In order for LGAs to be able to effectively and efficiently deliver social services to the people, Tanzania’s government policy paper states clearly that LGAs will adopt a fiscal decentralisation approach based on the principle that LGAs will have the financial discretionary power to generate local resources in addition to the adequate and unconditional resources that will be provided by the central government [4]. In other words, apart from receiving grants from the central government, the D by D policy provides autonomy to LGAs to generate their own resources and to plan how to utilise those resources for socio-economic and political development in their respective areas. However, our previous study, Frumence et al. [5] reported that LGAs in Tanzania lack adequate and reliable sources to generate their own resources to finance the delivery of health services in the councils. This situation has led LGAs to be entirely dependent on central government grants. Furthermore, the central government grants not only are inadequate to support local health needs and priorities, but they also are granted to LGAs based on various conditions, including a maximum regarding how much LGAs should budget. The aim of this paper is to examine how the dependency on central government funding in Tanzania affects health activities in Kongwa district council and the ways in which council health management teams cope with this situation.

### Overview of the funding of the district health system in Tanzania

On the mainland of Tanzania, LGAs are responsible for delivering public services, which include five grant-supported sectors: primary and secondary education; local health services; agriculture extension and livestock; water supply; and local road maintenance. It is estimated that, on average, three quarters of local government spending in Tanzania is for these sectors and the remaining one quarter is spent on other LGA functions such as refuse collection and administration. Local government expenditures also can be divided into recurrent expenditures, such as salary expenditures and other recurring operational costs, and development expenditures, such as spending on capital infrastructure. Recurrent public expenditures in Tanzania are further divided into wages and wage-related expenditures (Personal Emoluments, commonly abbreviated as PE) and non-wage expenditures (Other Charges, commonly abbreviated as OC) [6].

There are two major sources of funds provided by the central government to finance the delivery of health services by district health systems, for both recurrent and non-recurrent expenditures. They are basket funding (with several donors contributing to the basket) and block grants, which stem mainly from government generated revenues [7]. The health block grant and basket funds from the central government are allocated to councils using a needs-based formula. The formula considers four main factors: population (70%); poverty rate (10%); district vehicle route (distance), which is a proxy for the size of the area covered (10%); and mortality rate, for which under-five mortality is used as a proxy indicator (10%) [7,8].

Apart from block grants and basket funds from the central government, the district/council health systems also generate funds from other sources such as local taxes, cost sharing, community health funds (CHF), National Health Insurance Funds (NHIF) and user fees through cost sharing, as well as funds from other donors who do not contribute to the basket funds [7]. Generally, LGAs in Tanzania depend mainly on grants from the central government to finance public service provisions, including health services. Locally generated revenues cover less than 10% of the expenditures [9].

The central government has issued guidelines regarding how the district health system, through CHMT, should prepare the Comprehensive Council Health Plan (CCHP) before receiving funding from the central government. The activities included in the CCHP should reflect the priority areas of the Essential Health Package (EHP) issued by the central government through its Ministry of Health and Social Welfare (MoHSW). Six priority areas have been identified under the EHP, and for the CHMTs to be able to receive funds from the central government, they should, among other things, develop health interventions/activities that are within the areas identified by the EHP. The emphasis of the EHP is to ensure that health interventions cover the main diseases and health conditions responsible for the bulk of the disease burden in Tanzania. The six priority areas, ranging from the top to the lowest in that order, include reproductive and child health, communicable disease control, non-communicable disease control, treatment and care of other common diseases of local priority within the Council, e.g., eye disease and oral conditions, community health promotion/disease prevention, and the establishment/strengthening of organisational structures and institutional capacities for the improvement of health service management at all levels [10].

The reviewed literature [6,11] shows that there is a long process involved before basket funds can be approved for allocation to LGAs. The CHMT, through councils, submit the draft CCHP to the Regional Secretariat for comments and technical guidance before it can be approved by the Full Council (District Council). The approved CCHP is re-submitted to the Regional Secretariat, which then submits the CCHP for all the councils to the Prime Minister’s Office – Regional Administration and Local Government (PMO-RALG). The PMO-RALG will review the comments and recommendations of the Regional Secretariat and will consult with the MoHSW to obtain technical input and to ascertain whether the CCHP are in line with national objectives and performance indicators. Then, the MoHSW will provide feedback to the PMO-RALG; the guidelines state that there should be agreement between the MoHSW and the PMO-RALG regarding the recommendations for each council prior to the submission of the CCHP to the Basket Funds Committee. This committee, which consists of members from the donor community, and representatives from the MoHSW, the PMO-RALG and the Ministry of Finance, is responsible for the final approval of the amount of money that will be allocated to each LGA. However, according to government reports [6], there are other conditions that must be fulfilled before funds are disbursed to LGAs. In addition to an approved CCHP, the disbursement of the first quarter’s funds usually requires a submission of the financial accounts of the first six months and a technical report for the previous year. If these documents are not submitted on time, they may contribute to the delay in the disbursement of funds.

The block grant funds, which are mainly financed by the central government, are supposed to be released by it to LGAs once the CCHP has been approved. However, a previous study [5] has shown that disbursements of funds for other charges are also made late, primarily because block grants are dependent on the collection of national revenues.

## Methods

### Study setting

This study was conducted in Kongwa, which is one of two districts in the Dodoma region in which the health system project is being implemented. Dodoma region is situated in the central part of Tanzania. Kongwa was purposively selected as a typical rural district that has a moderate level of socio-economic development and is fairly accessible in terms of transportation and communication networks. Kongwa district borders the Manyara region to the north, the Morogoro region to the east, the Mpwapwa district to the south and the Dodoma rural district to the west. Administratively, the district has 14 wards, and according to the 2012 Tanzania National Census, the population of the Kongwa district was 309,977 [12]. The health care system in the Kongwa district is largely based on public health facilities, including one district hospital, three health centres and 27 dispensaries.

### Study design

A qualitative case study design was employed in this study in order to allow an in-depth and comprehensive exploration of how dependency on central government funding affects the implementation of health service delivery at the council level and how the councils cope with this situation in a real-life context [13]. As a case study, the selected council reflects the main impact caused by the late disbursement of central government funds to the LGAs and the strategies adopted by the council health management to cope with this situation.

### Selection of study participants

Data for this study were collected from members of the CHMT, who are the key decision-makers and planners of health services at the council level, and key officials of the LGAs, who are involved in the day-to-day operations of health services, because of their roles in the provision of health services at the council level. These officials were purposively selected because they were directly involved in making decisions as well as the actual implementation of the decentralised health services at the district council level. Specifically, 10 key informants who were directly involved in the planning, implementation and supervision of the decentralisation of health services at the council level were interviewed. Of these, seven were members of the Council Health Management Team, namely the District Medical Officer, the District Health Officer, the District Health Secretary and four heads of programs. The District Planning Officer (DPLO) was involved because he is in charge over all of the council plans and he is involved in developing a comprehensive council health plan. The District Treasurer (DT) was involved in the study because he is in charge of the finance department, and thus, is the chief advisor regarding financial issues at the council level, including health funds. Finally, the Chairperson of the Council Health Service Board (CHSB) was involved as a key informant because this board is responsible for discussing and amending district health plans and budgets, as well as identifying and soliciting funds to run the health services at the council level. One focus group discussion was conducted, which involved eight members of the CHMT.

### Data collection

Data collection for this study involved three main sources: interviews with key informants at the council level, focus group discussions with CHMT members and a review of key documents. Interviews with key informants and focus group discussions (FGDs) were carried out between January and February 2013. Interviews and FGDs lasted between approximately 60 and 90 minutes and 90 and 120 minutes, respectively. Interviews with key informants were conducted either at the offices of the respondents or in seminar rooms, as specified by the interviewees, while FGDs were conducted in a seminar room; all venues provided adequate privacy for the interviews and discussions to take place. Both interviews and FGDs were tape-recorded. Interview and FGD guides were developed and organised into different themes to guide the interviews and the discussion.

The guide was comprised of questions regarding the key informants’ knowledge about the reasons for the late disbursement of central government grants and funds to LGAs, the impact of late disbursement on the delivery of health services and the coping strategies adopted by the CHMT to deal with the late disbursement of government grants and funds for the last three financial years (2010/2011, 2011/2012 and 2012/2013). Both interviews and FGDs were conducted in Kiswahili to minimise language barriers and were later translated into English to facilitate joint analysis among all the authors. A number of key documents, particularly Kongwa district records, government policy papers and reports on decentralisation policy, were purposively selected and reviewed to provide a perspective regarding the mechanisms for the disbursement of central government grants and funds to LGAs.

### Data management

A daily review of data generated from the in-depth interviews and FGDs was conducted to ensure completeness and accuracy. Field assistants and researchers checked notes taken during data collection against audio-recorded information. They labeled audio-recorded cassettes and assigned unique codes to ensure easy identification of the respondent. Recorded information was then transcribed verbatim and the transcripts were filed electronically under unique codes.

### Data analysis

In this study, a thematic analysis was employed. Data were coded without necessarily fitting them into a pre-existing coding frame or the researcher’s analytic preconceptions. The transcribed data were analysed manually by the first author in collaboration with other authors by examining the transcripts, field notes and reviewed documents. Using an inductive approach, the authors identified the concepts that emerged and were strongly linked to the data themselves [14] and that described the phenomenon under investigation [15]. These concepts were further analysed to identify their similarities and differences; subsequently, they were grouped together to form more precise categories that were later organised into themes based on the research objective.

### Ethical issues

The Muhimbili University of Health and Allied Sciences approved the protocol for this study and the Dodoma region, the Kongwa district, and ward and village authorities granted their permission to undertake this study in the areas under their jurisdiction. Informed consent was obtained from all the key informants and FGD participants who participated in the study and they were informed of their right to withdraw from the study at any time. All the interviews and discussions were tape-recorded as well as recorded in notebooks with the permission of each participant, and the participants were informed that the results of the interviews and discussions will be kept confidential.

## Results

The analysis of the dependency of LGAs on central government funds has generated a wide range of issues. They can be classified into four main themes: Late disbursement of central government funds to LGAs, the reasons for late disbursement, the impact of late disbursement on district health activities, and the coping strategies of the LGAs in dealing with the late disbursement of funding from the central government. Supporting quotations from the study participants have been included to illustrate the messages being communicated.

### Late disbursement of central government funds to LGAs

The officials working with the local government authority at the district level and the council health management teams that were interviewed underscored that central government funds are the primary source of finances to run social and economic development activities in the local authorities. This concern was expressed by a local authority official:

“Remember that local government finances depend almost entirely on transfers of funds from the central government. As I said earlier, our own source of funds is very little” (KI 2).

When asked about the timely disbursement of funds from the central to the local government, our key informants (KI) reported that, comparatively, funds for personal emolument are disbursed to the LGAs on time and as per the budget approved by the national treasury. One KI had this to say:

“Usually we do not have problems regarding PE. The salaries are disbursed on time and as approved by the Central Establishment and Treasury” (KI 1).

However, information obtained from the majority of the KI and FGD participants revealed that funds for OC and basket funds are disbursed late. The health management teams usually develop comprehensive council health plans (CCHP) approximately between November and March, and the implementation of these plans begun in every new financial year, starting in July. Therefore, the CHMT is supposed to receive funds from the central government to enable it to start the implementation of the planned council health activities. However, our respondents reported that, in most cases, the central government is very late in disbursing money for other charges (OC), as expressed by one of the KI:

“We usually budget for OC funds, but the money is not disbursed on time. It is supposed to be released on a monthly basis, but we can wait for up to three months without receiving it, and sometimes, the funds are not released at all” (KI 2).

Key informants said that the basket fund is supposed to be disbursed by the central government every quarter. However, this is not done, as narrated by one of the KIs:

“The health department has the advantage of having the basket fund as another source of money. The activities supported by this fund are planned to start in July and September, as the first quarter, but sometimes, we do not receive the money until December” (KI 7).

Findings from the FGDs also pointed out that the central government has been delaying the release of funds for the implementation of various health activities at the council level.

“The other weakness regarding the disbursement of central government funds is that funds are delayed. For example, we may have planned to carry out a certain activity from January to March, but we don’t have the funds. A good example is the last financial year (2011/2012), during which the funds were disbursed at the end of the financial year” (FGD participant 6).

Findings from FGDs corroborated by records of disbursement of funds from central government to Kongwa district, showed that for the two consecutive financial years (2011/2012 and 2012/2013) the central government delayed the disbursement of funds to LGA for financing the health service activities as summarized in Table 1.

**Table 1:** Timing of disbursement of funds to LGA by the central government

**Financial year**	**Quarter**	**When funds were supposed to be disbursed to LGA**	**When funds were actually disbursed to LGA**
2011/2012	1	1^st^ July 2011	20^th^ November 2011
	2	1^st^ September 2011	20^th^ November 2011
	3	1^st^ January 2012	16^th^ April 2012
	4	1^st^ April 2012	16^th^ April 2012
2012/2013	1	1^st^ July 2012	28^th^ November 2012
	2	1^st^ September 2012	28^th^ November 2012
	3	1^st^ January 2013	20^th^ February 2013
	4	1^st^ April 2013	18^th^ May 2013

Source: Kongwa district records on the disbursement of funds from central government.

### The reasons for late disbursement

KIs and participants of FGDs were asked to give reasons regarding why the central government delays the disbursement of funds to LGAs. While some of the KIs and participants of the FGDs said that they were not aware of the reasons why this delay occurs, most of them mentioned two main reasons to which they attributed the delay. The first reason reported is that the government depends entirely upon revenue collection to generate funds for the allocation to LGAs for PE and OC, and usually, the revenue collected is not enough to cover PE and OC. The first priority after the collection of revenues is to ensure that salaries are paid on time; that is why the funds for PE are always released on time. KIs and participants in the FGDs reported that funds for OC seem to be a second priority in the government funding system:

*“Lack of adequate revenue collection makes it difficult for the central government to allocate funds for both PE and OC at the same. The first priority is salaries, followed by OC” KI 5*.

Information obtained from KIs, FGDs and reviewed literature [10] shows that the bureaucracy involved in the approval of basket funds prior to their allocation to LGAs is the main cause of delay for this particular type of funds. There are several organs involved in the approval of basket funds, including MoHSW, PMO-RALG, the donor community and the Ministry of Finance. One KI commented:

We (the council) have to write and submit a report to the ministry regarding how we have spent the previous allocation of basket funds. The report has to be approved by a number of committees before the ministry of finance releases the funds to us” KI 5.

### The impact of late disbursement on district health activities

Respondents had different views regarding the question of the impact of the late disbursement of funds on the implementation of council health activities. The main impact caused by the delay of the funds is the disruption of the work plan for various health activities in the council. The CHMT members who participated in this study underscored that they usually develop a work plan for all activities in a year. However, once funds are delayed from the central government, this automatically delays the implementation of many health activities.

“Primarily, the implementation of activities becomes inefficient as many activities pile up. When funds are disbursed, we find ourselves having a short time to implement the planned activities. You have to hurry up, which leads to a poor quality of health services, and finally, poor performance” (FGD participant 4).

Another FGD participant expressed the following concern:

In our work plan, we do have seasonal activities. For instance, during the rainy season, we have planned to sensitise community members on the elimination of mosquito breeding sites by removing any standing water which may be found around their houses or in the surrounding environment….If we have no money, this intervention cannot be done on time” (FGD participant 2).

### The LGAs’ strategies for coping with the late disbursement of funding by the central government

The KIs and participants of FGDs were asked to explain the strategies used by the CHMT in coping with the late disbursement of funds by the central government. Respondents mentioned three major strategies referred to here as sub-themes; namely, *borrowing money from other projects, obtaining supplies and other services on credit, and the use of cost sharing funds.*

#### ***Borrowing money from other projects***

One of the strategies used by the CHMT to cope with late disbursement of funds by the central government is to borrow money from other projects in order to continue with the implementation of health activities in the council. The CHMT reports to the director of the council, who is in charge of all project money in the council, and makes a request to borrow money from other accounts to continue with the delivery of health services while awaiting money from the central government. The director can only allow CHMT to borrow money from projects which have adequate funds to implement their activities for a long period of time in a year.

“In most cases, we do not get completely stuck in implementing health activities. We convene CHMT meetings and agree to consult the Council Director and borrow money from the accounts of other projects. We return the money once the funds are disbursed from the central government” (KI 1).

The same information was reported during FGDs with CHMT members:

“If the DED (District Executive Director) has money in other accounts and is approached by CHMT members, especially the DMO (district medical officer), he will lend us some money to continue with health activities such as the purchase of fuel to conduct supervisory visits to the health facilities” (FGD participant 1).

### Obtaining supplies and other services on credit

Apart from borrowing money from other accounts, CHMT members who participated in this study reported that they also obtain supplies and repair vehicles on credit to cope with the late disbursement of central government funds. They said that the CHMT has entered into contracts with suppliers of fuel and vehicle spare parts and automobile repair workshops, some of whom are willing to continue to supply the required services and supplies on credit, and to receive reimbursement once the CHMT receives the funds from the central government.

Yeah! We might not have funds, but since we have a contract with suppliers, we buy different items and services on credit, including vehicle repairs, fuel for vehicles and food for patients. Then, we pay them once we receive money from the central government (KI 1?).

However, some KIs cautioned that there are limitations to their ability to purchase many supplies and services from suppliers in the absence of immediate payment. In rare cases, misunderstandings are caused by distrust between suppliers and the office of the DMO resulting from delays in the settlement of the debts.

### Use of cost sharing funds

Some of the KIs that were interviewed and participants of FGDs claimed that, on rare occasions, and especially when there is an urgent need; funds generated by cost sharing can be used to cover the costs to purchase drugs and medical supplies in cases in which the central government has delayed the disbursement of funds to the CHMT. The respondents further reported that, although cost sharing was specifically designed to be a strategy to generate funds for health systems to supplement the funds from the central government, cost sharing sometimes becomes the only source of funds that is available to facilitate the provision of health services, including the purchase of drugs, medical supplies and equipment. However, this has been adopted as a temporary solution while awaiting the release of funds by the central government to the CHMT.

“CHMT have other options to cope with late disbursement of funds by the central government, such as the use of cost sharing funds to purchase drugs and other supplies, especially when there is a really urgent problem” (KI 1).

## Discussion

Studies have documented the challenges and barriers facing decentralised health systems in Sub-Saharan Africa in general [16], and Tanzania, in particular [5]. However, this is one of the few papers in Tanzania and beyond that has documented the strategies used by the decentralised health systems to cope with the identified challenges or barriers that hinder the smooth operation of health service delivery in their localities, and particularly, the challenges resulting from the late disbursement of central government funds.

The local government authorities in Tanzania are largely dependent on central government funding to run their socio-economic activities, including health activities. The worst issue arising from this dependence is that the transfer of funds is characterised by late disbursement to the decentralised structures, including health systems. The bureaucracy involved in the approval of funds for LGAs has been reported to contribute partly to this situation.

This study has revealed three major strategies adopted by the council health management team to cope with the late disbursement of funds by the central government. The first and most common strategy is obtaining supplies and other services on credit from suppliers who have been contracted by the LGAs. The second strategy is to borrow money from other projects, which have funds to implement their activities for a long period. These are the projects that operate in the district under the District Executive Director (DED). The third strategy is the use of money generated from cost sharing. All these strategies enable the CHMT to purchase fuel, drugs, basic medical supplies and any other necessary items and services so as to maintain the continuous operation of health services in the council.

Concerning the bureaucratic procedures involved in the approval of funds for local government authorities, it has been noted that it is not a new phenomenon in Tanzania. This phenomenon also has been reported in other developing countries, including Botswana, in which local authorities must face a slow and cumbersome procedure before funds are approved by the ministry of finance and development planning [17].

This study has shown that the trust and formalised contracts between the district health system represented by the CHMT and suppliers has strengthened the partnership between those two organisations (LGAs/CHMT and suppliers). This mutual understanding has facilitated the constant flow of supplies such as fuel and equipment necessary to continue health service delivery even during times when the CHMT does not have the money to make payments immediately. These are efforts that are self-initiated by the CHMT and council management to ensure that health service delivery is not interrupted, but they are not officially recognised by the central government as methods to finance the delivery of health services in the councils. Similarly, findings from Ghana indicated that, when district health systems do not have funds from the central government to run health activities, the district health managers enter into agreements with local suppliers who are willing to supply fuel and other important medical supplies on credit, and that this strategy has played a significant role in allowing health services to run smoothly [2].

In practice, cost sharing in Tanzania is a strategy that was developed pursuant to health sector reforms to generate funds for health systems supplementing central government funds, ideally making it possible to provide a high standard of health services to the community and to ensure a constant and adequate supply of drugs [18,19]. In this study, use of money collected through cost sharing has been reported as a strategy for CHMT to cope with late disbursement of central government funds. A study of cost sharing in urban and rural districts in Tanzania found that cost sharing accounts for only 4% of the total LGA expenditures on health services, which indicates that the small amount of revenue generated only supplements funding by the central government [18]. This demonstrates that the central government, particularly in low income countries like Tanzania, still plays a major role in providing financial support to ensure the effective implementation of health services to the population at large.

### Implications for decentralised health systems

In the technical discussion report regarding the role of local governments in health development, WHO has underscored the role of central governments, through ministries of health and other related ministries and agencies involved in health development, especially in strengthening health systems and in generating both human resources and non-human resources such as finances. In so doing, the government ensures that decentralised health systems achieve their expected goals to improve health as well as to reduce existing health inequalities and to attain equity in health care financing [20,21]. Since the late 1990s, the MoHSW, through decentralisation and sector-wide approaches within the health sector, has developed various guidelines endeavouring to improve health services for the population. These guidelines include how to prepare the CCHP and arrangements for the disbursement of basket funds and block grants to LGAs [10]. However, these efforts, which are aimed at improving performance in the management of the decentralised health systems and enhancing decentralisation, will not bear any fruit if the central government continues the late disbursement of funds to LGAs. This study calls for the central government to eliminate bottlenecks in the transfer of resources, particularly funds flowing from the national level to LGAs, in order to enable the decentralised health systems to achieve their expected goals.

### Limitations of the study and suggestions for further work

The selection of one district for this study limits the generalisability of the findings. However, this district was purposively selected as a typical rural district with a moderate level of socio-economic development, and that is fairly accessible in terms of transportation and a communications network. This study also did not conduct an enquiry from the central government side on issues that interfered with timely disbursement to the districts so as to get experiences and national perspective on this issue. However, notwithstanding these limitations, findings from this district have provided insights into what is happening in the decentralized health systems in the country, particularly given the fact that the way CHMTs operate in delivering health services is similar throughout the country. Nonetheless, it is suggested that another study with a large sample covering more districts and regions is needed to provide a broader picture of how the late disbursement of funds from the centre to LGAs affects health service delivery and explore the magnitude of such impact in the decentralized health system in the country and how CHMTs cope with the situation.

## Conclusion

Decentralised health systems are very dependent on resources from the central government to run health services. The late release of central government funds to district health systems undermines health service delivery and hinders the effective implementation of the health activities identified in the comprehensive council health plans. Despite the fact that the late disbursement of funds for the implementation of health activities at the district level demoralises CHMTs and other health workers, CHMTs have exerted a great deal of effort to design the best strategies to cope with the situation and to enable the health system to continue operating. Because the central government, through MoHSW and PMO-RALG, has developed various guidelines to facilitate the disbursement of funds to decentralised structures in the country, including health systems, it is the responsibility of the same central government to ensure that those guidelines do not result in administrative bottlenecks. Rather, it should become a facilitating agent for the timely release of funds to local government authorities. Because those funds are an important resource for the achievement the objectives of the policies aimed at improving the health of the population in Tanzania, policy makers at the national level should devise mechanisms to ensure that the timely disbursement of funds to the LGAs is a top priority in their day-to-day operational management. Without an effective and efficient mechanism for the transfer of resources from the central government to LGAs, the expected goal of the decentralised health system to improve the health of the population will not be achieved.

## Abbreviations

CCHP: Comprehensive council health plan; CHF: Community health fund; CHMT: Council health management team; CHSB: Council health services board; D-by-D: Decentralization by devolution; DPLO: District planning officer; DT: District treasurer; EHP: Essential health package; FGD: Focus group discussion; KI: Key informant; LGAs: Local government authorities; NHIF: National health insurance funds; OC: Other charges; PE: Personal emoluments; PMORALG: Prime Ministers’ office, regional administration and local government; RHMT: Regional health management team; URT: United Republic of Tanzania.

## Competing interests

The authors declare that they have no competing interests.

## Authors’ contributions

GF planned the study, supervised data collection and analysed the data, and prepared the first draft of this manuscript. TM and MM contributed to the design of the study and analysis of data, and commented on the first and subsequent drafts of this manuscript. AKH contributed to the design of the study and commented on the first and subsequent drafts of this manuscript. All authors read and approved the final manuscript.

## Pre-publication history

The pre-publication history for this paper can be accessed here:

http://www.biomedcentral.com/1472-6963/14/39/prepub
